# Encapsulation of sulfur with thin-layered nickel-based hydroxides for long-cyclic lithium–sulfur cells

**DOI:** 10.1038/ncomms9622

**Published:** 2015-10-16

**Authors:** Jian Jiang, Jianhui Zhu, Wei Ai, Xiuli Wang, Yanlong Wang, Chenji Zou, Wei Huang, Ting Yu

**Affiliations:** 1 Nanyang Technological University—Nanjing Tech Center of Research and Development, Nanjing Tech University, Nanjing 211816, China; 2 Division of Physics and Applied Physics, School of Physical and Mathematical Sciences, Nanyang Technological University, Singapore 637371, Singapore; 3 State Key Laboratory of Silicon Materials, Key Laboratory of Advanced Materials and Applications for Batteries of Zhejiang Province, and School of Materials Science and Engineering, Zhejiang University, Hangzhou 310027, China; 4 Key Laboratory of Flexible Electronics (KLOFE), Institute of Advanced Materials (IAM), Nanjing Tech University (NanjingTech), 30 South Puzhu Road, Nanjing 211816, Jiangsu, China; 5 Key Laboratory for Organic Electronics and Information Displays and Institute of Advanced Materials (IAM), Nanjing University of Posts & Telecommunications, 9 Wenyuan Road, Nanjing 210023, Jiangsu, China; 6 Department of Physics, Faculty of Science, National University of Singapore, Singapore 117542, Singapore

## Abstract

Elemental sulfur cathodes for lithium/sulfur cells are still in the stage of intensive research due to their unsatisfactory capacity retention and cyclability. The undesired capacity degradation upon cycling originates from gradual diffusion of lithium polysulfides out of the cathode region. To prevent losses of certain intermediate soluble species and extend lifespan of cells, the effective encapsulation of sulfur plays a critical role. Here we report an applicable way, by using thin-layered nickel-based hydroxide as a feasible and effective encapsulation material. In addition to being a durable physical barrier, such hydroxide thin films can irreversibly react with lithium to generate protective layers that combine good ionic permeability and abundant functional polar/hydrophilic groups, leading to drastic improvements in cell behaviours (almost 100% coulombic efficiency and negligible capacity decay within total 500 cycles). Our present encapsulation strategy and understanding of hydroxide working mechanisms may advance progress on the development of lithium/sulfur cells for practical use.

## Abstract (web summary)

Development of lithium-sulfur cells is hindered by many problems such as the dissolution of polysulfides. Here, the authors use thin nickel-based hydroxide layers to entrap both sulfur and intermediate polysulfides in the cathode, which mitigates the problems leading to high battery performance.

## Graphical abstract

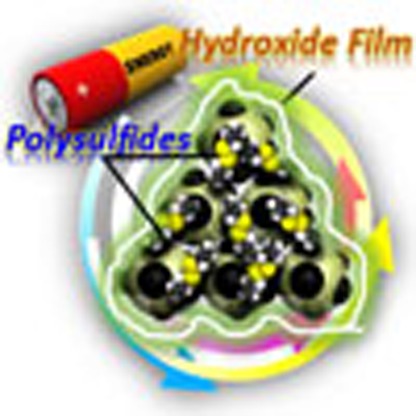

Lithium/sulfur (Li/S) cells are promising energy storage devices to power electric vehicles for long-distance driving (>300 miles per charge) due to their upper theoretical energy density and lower price in comparison with currently used Li-ion cells^1^,^2^,^3^. According to charge/discharge voltage profiles or electrolytes applied in Li/S cell systems, the cathode materials can be generally categorized into two types: (1) the elemental sulfur (aggregated cyclo-octasulfur S_8_) and (2) a series of sulfur-derived composites^4^. Elemental S_8_ owns overwhelming advantages over the synthetic thionic composites. On one hand, it is environmentally benign and abundant in nature, hence readily available and fairly cheap in markets; on the other hand, when coupled with Li metal anode, it operates at a safer voltage of ∼2.15 V (versus Li/Li^+^) compared with conventional Li-insertion compounds (∼3–4.5 V versus Li/Li^+^), and offers a higher energy density than thionic counterparts^4^,^5^,^6^,^7^. The S_8_ can exhibit a total theoretical capacity of 1,672 mAh g^−1^ when undergoing an overall redox reaction of S_8_+16Li^+^+16e^−^↔8Li_2_S (ref. 8). The corresponding energy density reaches as high as ∼2,567 Wh kg^−1^, more than sixfold that of commercial LiCoO_2_/C cells (∼387 Wh kg^−1^)^9^.

The development of Li/S cells based on pure S_8_ cathode, however, is impeded by several challenges unfortunately. Primarily, both S_8_ and the discharged end products Li_2_S_2_/Li_2_S are insulators^10^. Particularly noteworthy is that, Li_2_S is an extremely poor electrically/ionically conducting material with electrical conductivity of ∼10^−30^ S cm^−1^ and Li^+^ diffusivity of ∼10^−15^ cm^2^ s^−1^, which inevitably poses inferior cell kinetics on charge transfer and low utilization efficiency of S_8_ (ref. 3). Next is the undesired self-discharge issue in Li/S cells. Unlike LiCoO_2_/C cells with stable passivation layers covering on electrode interfacial surfaces, in Li/S cell system, the S_8_ cathode exposed in electrolyte under a fully charged state tends to react with Li^+^ gradually, convert to polysulfide species and dissolve into the electrolyte, which eventually results in a static energy decrease in cell capacity^11^. Last but the foremost, along repeated charge/discharge procedures, the unavoidable dissolution and loss of intermediate polysulfides (Li_2_S_n_, *n*=3–8), together with their notable ‘shuttle effects' between the anode and cathode, will inevitably lead to the formidable issues of severe capacity decay, low coulombic efficiency and limited cyclic life^12^,^13^,^14^.

The long-lasting and stable cyclic behaviour is undoubtedly the fundamental prerequisite for future commercialization of Li/S cells. To prolong the cyclic lifetime, a worldwide-recognized approach is to wrap the active S_8_ with an effective physical barrier so as to solidly confine the soluble intermediate polysulfides in the cathode region^3^,^15^,^16^,^17^,^18^,^19^,^20^,^21^,^22^,^23^,^24^,^25^,^26^,^27^. Several S_8_ encapsulation avenues have been presented to date, normally by the use of carbon materials (typically like two-dimensional graphene), conductive polymers or diverse inorganic coating layers^15^,^16^,^17^,^18^,^19^,^20^,^21^,^22^,^23^,^24^,^25^,^26^,^27^. Though using intrinsically mesoporous carbons is taken for granted as an ideal strategy to restrict polysulfide molecules in cathode matrices^15^,^16^,^17^,^18^,^19^, actually weak interactions between non-polar carbon materials and Li_2_S_n_ reduce the ability to bind and entrap these soluble polar species^20^. As a consequence, only use of carbon materials can benefit the cell kinetics/reversibility and alleviate the capacity fading but still fail to resolve the short cyclic problem. Though soft conductive polymers or organic frameworks are also conceived as preferred and competitive inhibitors to hinder the polysulfides shuttling, their behaviours on elongating the cell lifetime are yet far from perfect^21^,^22^,^23^,^24^. By contrast, encapsulations realized via inorganic species (for example, S_8_–TiO_2_ yolk–shell nanoarchitectures) hold great potential to build long-term cyclic Li/S cells^25^,^26^,^27^. Nevertheless, such state-of-the-art core–shell nanohybrids yet show little promise in broad commercial use owing to limitations such as low tap density and high surface areas of nanomaterials^28^, excess interparticle boundaries contained in electrode systems^29^ and so on. Therefore, to overcome the major cyclic constraint and bring Li/S cells a step closer to commercialization, seeking for reliable ‘armors' fit for S_8_ cathode and an efficient encapsulating strategy applicable to bulks (∼20 μm scale) for higher volumetric energy is urgently pursued.

Herein, we demonstrate the feasibility of adopting semiconducting nickel nitrate hydroxide (Ni_3_(NO_3_)_2_(OH)_4_, denoted as NNH), one type of thin-layered α-Ni(OH)_2_ often applied in nickel–metal hydride cells and supercapacitors, as a novel and effective encapsulation material for S_8_ cathode. More than a durable physical shell, NNH is capable to irreversibly react with Li^+^ in initial tens of cycles, turning into reliable (Li, Ni)-mixed hydroxide compounds with a combination of good Li^+^ permeability/accessibility and copious functional polar/hydrophilic groups (for example, hydrophilic groups, surface hydroxyl groups and so on) existing in cathode systems^12^,^27^. In this work, we choose the simple hybrid of S_8_@carbon black (S_8_@CB; with a central dimension of ∼20 μm), as a paradigm to investigate the electrochemical functions of NNH. By using such a smart core–shell S_8_@CB@NNH hybrid as the cathode, we eventually achieve drastic improvements in capacity retention (almost ∼500% capacity rise when compared with the case of bare S_8_@CB) and long-term cyclic stability (negligible capacity decay within total 500 cycles). Our present work may open up a feasible and effective concept of using thin-layered transition metal hydroxides as a promising class of encapsulation materials to build better Li/S cells.

## Results

### Synthesis and characterization

**Figure 1:**
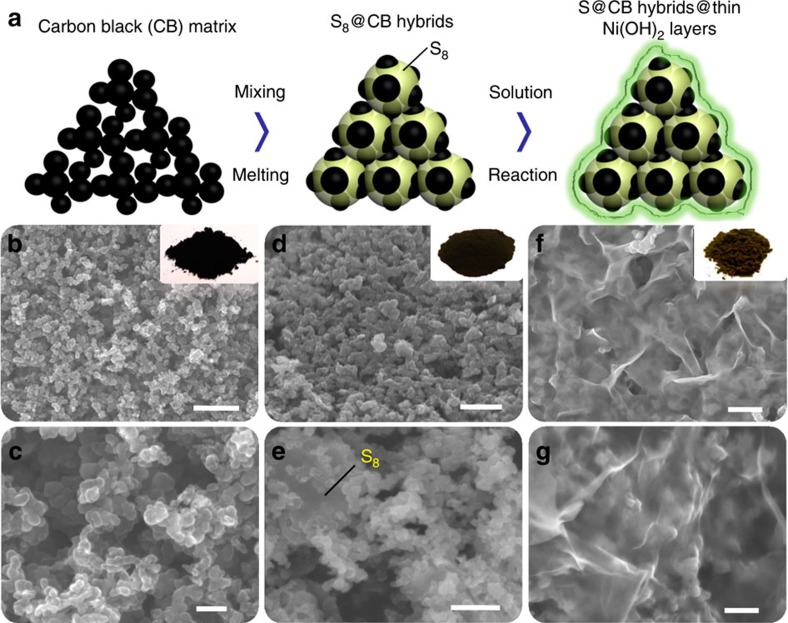
Schematic and s.e.m. images showing the fabrication of S_8_@CB@NNH hybrids. (**a**) A general schematic for the entire fabrication procedures. Basic s.e.m. observations towards (**b**,**c**) CB powders, (**d**,**e**) S_8_@CB intermediates and (**f**,**g**) S_8_@CB@NNH hybrids. The inset pictures correspond to their optical images, respectively. Scale bars, 200 nm (**b**,**d**), 50 nm (**c**,**g**) and 100 nm (**e**,**f**).

The entire fabrication of core–shell S_8_@CB@NNH bulky hybrids was schematically shown in Fig. 1a (see Methods section for details; basic characterizations on NNH are involved in Supplementary Fig. 1). In brief, the preparation of S_8_@CB particles was achieved by impregnation of molten S_8_ into CB matrices. The choice of CB powder, which has been long commercialized in cell technologies, as the S_8_ carrier is due to its large surface-to-volume ratios, excellent electrically conducting properties and far lower cost than other counterparts like advanced mesoporous carbons, graphene and carbon nanotubes and so on. Another notable reason is that there are numerous nanosized cavities (Fig. 1b,c) distributed in between CB nanoparticles, as reflected by the pore-size distribution plot (Supplementary Fig. 2). These interspaces (randomly distributed in a wide range of ∼1–50 nm) can not only supply abundant ‘reservoir' places to accommodate S_8_ but also help to downsize the S_8_ bulks into nanoparticles for preferable cell kinetics. Figure 1d,e shows typical scanning electron microscopy (SEM) observations on S_8_@CB powders. During the melting treatment, the S_8_ powders have definitely fused into a liquid state, penetrated/embedded into certain cavities and merged together with CB-conducting agents. The size for entire bulks is centred at ∼20 μm (Supplementary Fig. 3), while the diameter of individual S_8_@CB subunit lies in a range of ∼20–100 nm. The following procedure was undertaken at a low temperature of 95 °C in an aqueous solution where the S_8_@CB hybrids are uniformly packaged by NNH. The reaction of Ni salts with ammonium (NH_4_OH) yielded from the progressive hydrolysis of hexamethylenetetramine (C_6_H_12_N_4_+10H_2_O→6HCHO+4NH_4_OH) guarantees an intact encapsulation of NNH layers on each S_8_@CB particle. Fresh S_8_@CB@NNH products are then washed by centrifugation and dried at 60 °C in an electronic oven. S.e.m. observations (Fig. 1f,g) reveal that the as-formed S_8_@CB@NNH hybrids possess a well-defined core–shell configuration; all of the nanosized S_8_@CB subunits have been intimately packaged within the layered film structures. Also, the stepwise evolutions of S_8_@CB@NNH are further confirmed in parallel using energy dispersive X-ray spectroscopy (EDX) to monitor the entire fabrication flow (see details in Supplementary Fig. 4).

**Figure 2:**
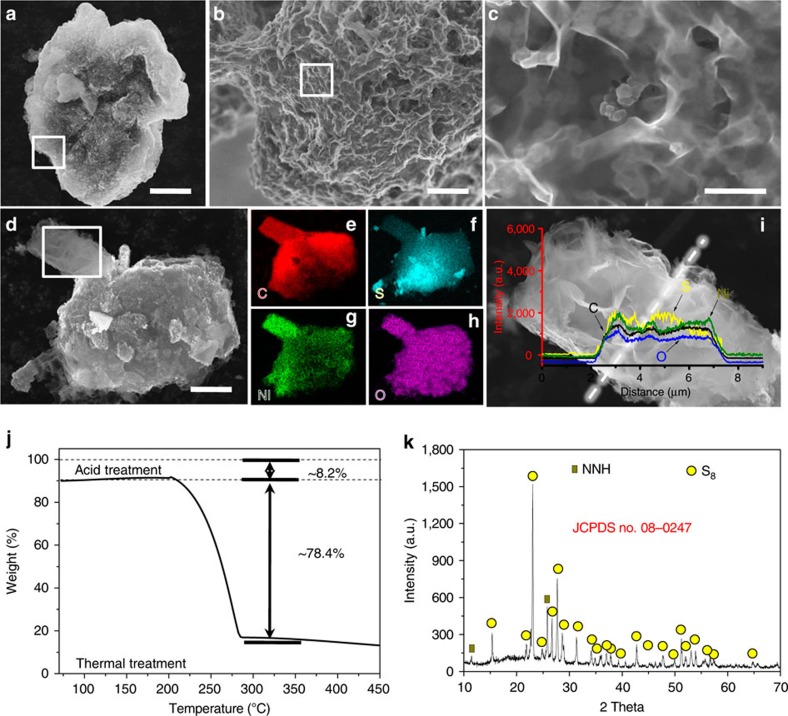
Characterizations of the S_8_@CB@NNH bulky hybrids. (**a**–**d**) S.e.m. images of S_8_@CB@NNH hybrids. (**e**–**h**) EDX elemental mapping records towards one typical S_8_@CB@NNH bulky sample, and (**i**) a line-scan analysis on a selected section of this hybrid product. (**j**) A plot recording weight losses of S_8_@CB@NNH products during an acid immersion treatment combined with a TG measurement. (**k**) XRD pattern of synthesized S_8_@CB@NNH powders. Scale bars, 10 μm (**a**,**d**), 1 μm (**b**) and 100 nm (**c**).

A special focus is then put on the ultimate products of S_8_@CB@NNH. Figure 2a–d shows their basic s.e.m. observations. The large-area SEM detection (Fig. 2a) illustrates that the self-assembled S_8_@CB@NNH product owns a bulky structural feature (∼20 μm upward in width) and an interesting heart-shaped geometric profile. No naked areas are observed on bulky S_8_@CB@NNH hybrids, ascertaining our success in perfectly packaging S_8_@CB into NNH shells. Close-up s.e.m. images (Fig. 2b,c) furthermore uncover their detailed structural information. Definitely, every S_8_@CB nanoparticle unit has been overall and closely wrapped with NNH ‘armors', as also confirmed by transmission electron microscope (TEM) image in Supplementary Fig. 5. Note that our used encapsulating material is supple enough to conform well to arbitrary body shapes of introduced precursors, enabling a tight and intact encapsulation of involved S_8_@CB powders. Moreover, the NNH films are extremely thin (the thickness for one NNH layer is only ∼7 nm, as evidenced by pioneering works)^30^,^31^; TEM and s.e.m. observations (Supplementary Figs 1e,f and 6) further reveal the distribution of pore defects on NNH surface. Such a thin-film characteristic, together with intrinsic porosity properties (for example, micropores/mesopores on NNH or in between these overlaid structures) may allow Li^+^ to move readily across the outer protective layers, pledging the expedite ionic access/permeation to inner places of entire cathode. Figure 2e–h displays the EDX elemental mapping records towards one bulky S_8_@CB@NNH product (see its SEM morphology in Fig. 2d). The mapping images visually declare a homogeneous dispersion of C, S, Ni and O atoms in this hybrid. In addition, we purposely carry out a line-scan analysis on a selected section of S_8_@CB@NNH (Fig. 2i). Though there is a visible thick layer of NNH covering this region, the involved S element still takes up the largest proportion (rather than Ni or O element) and stays evenly distributed. To clarify the actual contents of C, NNH and S_8_ in S_8_@CB@NNH hybrids, the powder samples thus undergo an acid immersion treatment followed by a thermogravimetric (TG) measurement (see experimental details in Methods section). The stepwise weight losses recorded in Fig. 2j reflect the detailed compositions of S_8_@CB@NNH, containing ∼8.2% of NNH, ∼13.4% of CB and ∼78.4% of active S_8_. Figure 2k shows the X-ray powder diffraction (XRD) pattern of S_8_@CB@NNH. In addition to distinguishable signals from NNH and amorphous CB, all other strong diffraction peaks stem from the crystalline S_8_ (JCPDS no. 08-0247).

### Electrochemical performance

**Figure 3:**
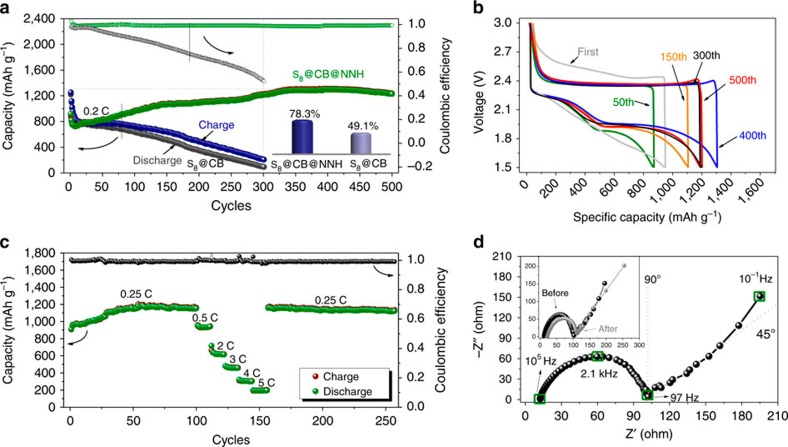
Cell performance of the S_8_@CB@NNH hybrids. (**a**) Long-term cyclic testing of S_8_@CB@NNH and S_8_@CB at a current rate of 0.2 C in a potential window of ∼1.5–3 V. The inset shows their utilization ratio of active S_8_. (**b**) Charge/discharge voltage profiles, (**c**) programmed cyclic responses and (**d**) electrochemical impedance spectrum of S_8_@CB@NNH cathode.

To evaluate the electrochemical properties of S_8_@CB@NNH hybrids, the as-synthesized electrodes are initially subjected to a long-term cyclic test in a potential window of ∼1.5–3 V at a constant current rate of 0.2 C (335 mA g^−1^; 1C=1,672 mA g^−1^). To verify and highlight the cooperative functions of NNH, bare S_8_@CB cathodes are also measured underneath the same conditions for comparison (Fig. 3a). The S_8_@CB cathode, as a whole, exhibits poor electrochemical behaviours including rapid decay in both specific capacity and coulombic efficiency and short cyclic life period. The discharge capacity on the first cycle achieves 1,345 mAh g^−1^ but it declines ceaselessly to a low capacitive value (less than ∼200 mAh g^−1^ after 300 cycles). On cycling, an increasing divergence between the charge and discharge capacity highly suggests the severe degradation on the coulombic efficiency. At the 300th cycle, only a coulombic efficiency value of ∼52% is sustained. The drastic decrease in capacity/coulombic efficiency would be mainly attributed to formidable kinetic issues in Li/S cell systems. A fraction of yielded polysulfide molecules that should be trapped in carbon reservoirs gradually migrate out of the cathode, and they may not be reversibly used again^32^. Even worse, undesired side reactions between these highly reactive polysulfide anions and electrolyte solvents would furthermore speed up the capacity losses^33^. By sharp contrast, the S_8_@CB@NNH cathode shows far better charge/discharge performance over 500 cycles. Among initial few cycles, the capacity of S_8_@CB@NNH in the beginning drops from the initial ∼968 mAh g^−1^ to a bottom level of ∼786 mAh g^−1^ (ninth cycle), which is mostly due to a delayed electrolyte infiltration into a well-capsulated structure. In subsequent 100 cycles (from the 50th to 150th cycle), the output for S_8_@CB@NNH-based cells rises progressively (since more S_8_ becomes activated) until an electrochemical equilibrium state is built. About 150 cycles later, the capacity grows slowly to a maximum reversible value of ∼1,326 mAh g^−1^ (410th cycle) and maintains stabilized at ∼1,250 mAh g^−1^ to the end of cycling (no similar capacity-rise phenomena happen on S_8_@CB cathodes). Capacitive growth in this stage may be attributed to unavoidable structural fatigue/damage of outer layers because film breaking may provide new open-up places and accordingly more available routes for Li^+^ to reach inner deep regions wherein the S_8_ is deadly trapped. Till the 300th cycle, the S_8_@CB@NNH cathode still stably outputs a capacity of ∼1,164 mAh g^−1^, almost six times greater than that of S_8_@CB. Also notice that unlike S_8_@CB-based cells, there is actually no capacity fading for S_8_@CB@NNH cathodes within prime 410 cycles. The coulombic efficiency always stays beyond ∼98%, indicative of outstanding electrochemical reversibility of S_8_@CB@NNH cathodes. Assuming the utilized S_8_ is fully activated and contributes a theoretical capacity, the utilization ratio of active S_8_ (calculated based on the maximum capacity in cycling) reaches up to ∼78.3%, with a substantial rise by 59.5% in contrast with that in S_8_@CB case (49.1%). Above evidences fully confirm that our facile encapsulation of NNH indeed plays a positive role in inhibiting the outward diffusion of long-chain soluble polysulfides from the cathode, and thus greatly promoting the long-cyclic cell performance.

With the goal of attaining valuable insights into the cyclic information, we carefully analyze the charge/discharge voltage profiles of S_8_@CB@NNH cathode. For better comparisons between the pristine S_8_@CB and final S_8_@CB@NNH hybrid on cell performances, additional charge/discharge profiles of S_8_@CB have also been provided (Supplementary Fig. 7). Figure 3b evidently shows that in the first electrochemical process, only a distorted discharge curve (rather than the smooth and distinct plateaus of bare S_8_@CB) is recorded. However, the discharge profiles lying at the 50th and subsequent cycles change a lot. Two main plateaus are present obviously, corresponding to the reduction of S_8_ into complex solubilized polysulfides (for example, Li_2_S_8_, Li_2_S_6_, Li_2_S_4_ and so on) over a potential range of ∼2.1–2.3 V and ultimate formation of solid-state Li_2_S_2_/Li_2_S at ∼2.0 V. Plateaus in later charge profiles are related to converse reactions from lithium sulfides to metastable polysulfides, and finally backward to the primitive S_8_. The appealing capacity-rise phenomenon is also recognized. The discharge plateau nearby ∼2.0 V extended gradually along with increased cyclic numbers, signifies that certain enhanced capacities are tightly correlated with complex conversions between highly ordered polysulfides and short-chain Li_2_S_2_/Li_2_S. We consider that the inner deep dispersions of active S_8_ and postponed electrolyte penetration led by outer enclosure of NNH multi-layers may well account for the presence of this unique capacitive activation. Apart from that, frequent Li^+^ migrations would endow exterior NNH layers with tiny micropore/mesopore architectures, quite favourable for Li^+^ diffusion; as a consequence, better utilization of S_8_ actives and easier conversion reactions of Li_2_S_4_/Li_2_S can be observed due to progressively enhanced electrochemical kinetics. Rate capabilities are further estimated by a cyclic test under programmed current densities (Fig. 3c). Corresponding charge/discharge voltage profiles at varied current rates are also displayed in Supplementary Fig. 8. The cell exhibits a stable cyclic behaviour at each current speed, with all coulombic efficiencies nearly approaching ∼100%. Allowing for sufficient electrode activation, the S_8_@CB@NNH cathode preliminarily suffers from continuous 100 cycles under a constant current rate of 0.25 C, with a delivered capacity of ∼1,153 mAh g^−1^ (at the 100th cycle). Afterwards, the cell runs sequentially at 0.5, 2, 3, 4 and 5 C, enabling an output discharge capacity of ∼943, ∼610, ∼467, ∼289 and ∼195 mAh g^−1^, respectively. This electrode rate performance is much superior to those in previous examples having similar ‘S_8_-in-carbon matrix' configurations^34^,^35^,^36^. Even when the current rate suddenly switches back to 0.25 C, an exceptional specific capacity of 1,149 mAh g^−1^ is able to be retained (almost ∼100% recovery ratio in stored capacity). Figure 3d presents the electrochemical impedance spectrum of assembled S_8_@CB@NNH cells at an open-circuit voltage. The hybrid electrode of S_8_@CB@NNH, albeit with an exterior package of NNH films, shows a comparable semicircle diameter to bare S_8_@CB cathodes among the high-frequency region (Supplementary Fig. 9). There are no considerable differences in terms of charge-transfer resistance (*R*_*ct*_), representing that such incorporation of NNH thin layers would not have a great effect on charge transfer. Moreover, little change is observed on *R*_*ct*_ impedance (see the inset in Fig. 3d) between original cells and the ones after 400 fatigue cycles, once again ensuring the good electrochemical stability of S_8_@CB@NNH cathode.

### Working principles of NNH for prolonged Li/S cells

**Figure 4:**
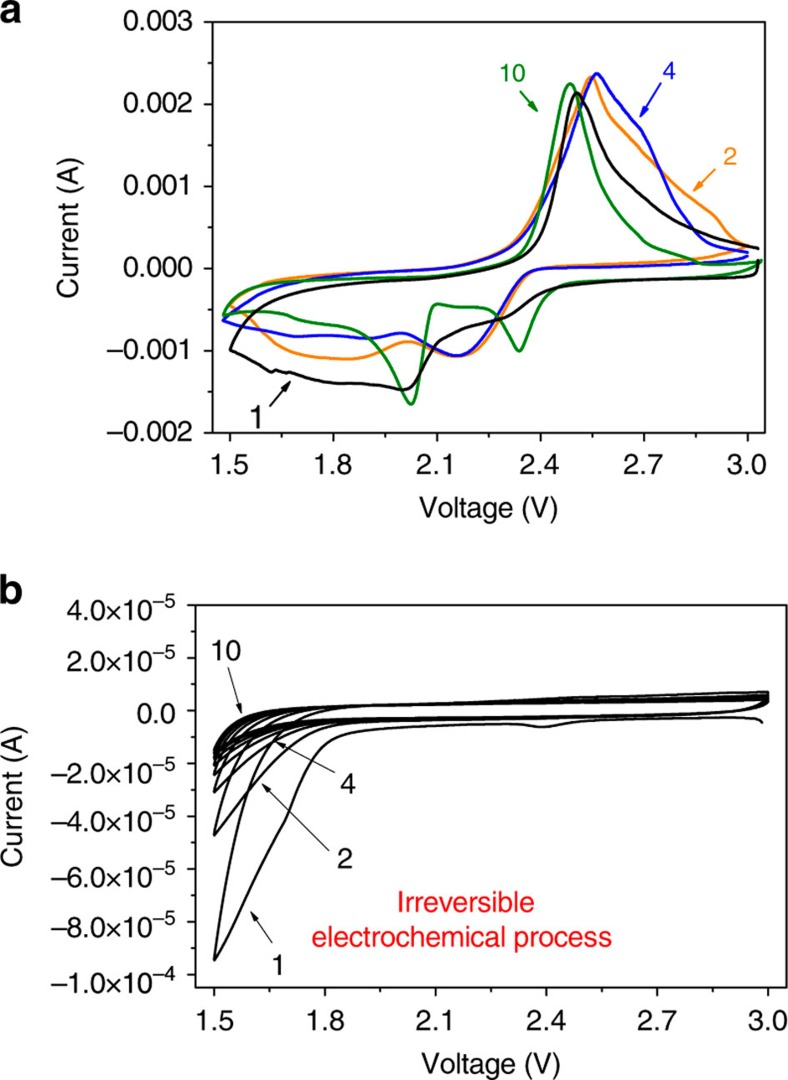
Electrochemical characterizations of the S_8_@CB@NNH cathode. CV testing of (**a**) S_8_@CB@NNH cathode and (**b**) CB@NNH under a scan rate of 50 μV s^−1^.

To make certain the change on discharge voltage profiles aforementioned, a cyclic voltammetry (CV) test at a slow scan rate of 50 μV s^−1^ is conducted (Fig. 4a). Besides reduction peaks that relates to the transformation of Li polysulfides to Li_2_S_*x*_, there seems other broad current responses emerging when cells are first scanned from 3.0 to 1.5 V. This unusual phenomenon may be greatly associated with undesired electrode polarizations/potential sluggish due to the presence of NNH outer films that would retard the electrolyte penetration and Li^+^ transfer. In subsequent second and fourth cathodic scans, the intensity of these current signals is gradually diminished. Whereas, in the 10th scan, two well-defined reduction peaks above ∼2.0 V are present, and very little current trace in a potential scope of ∼1.64–1.8 V is noticed. We suppose that these current signals appearing in the low potential range (<1.85 V) should be linked to electrochemical interactions among the electrolyte, Li^+^ and NNH. To prove this consumption, a CV scan towards the CB@NNH hybrids (without active S_8_) under the same electrochemical conditions is performed (Fig. 4b). In the initial CV scan, there emerges a strong irreversible current peak (its intensity is still negligible when compared with that for S_8_-contained cases) located at the position of ∼1.5–1.86 V whose potential scope is highly in line with our records in Fig. 4a. This result may properly support our above estimation. With the increase of CV scans, the intensity for such current signals tends to decrease gradually and at last almost vanishes over 10 cycles, which implies the termination of these irreversible reactions. This electrochemical process may lead to the vast generation of complex Li, Ni-mixed hydroxides on S_8_@CB particle surface.

**Figure 5:**
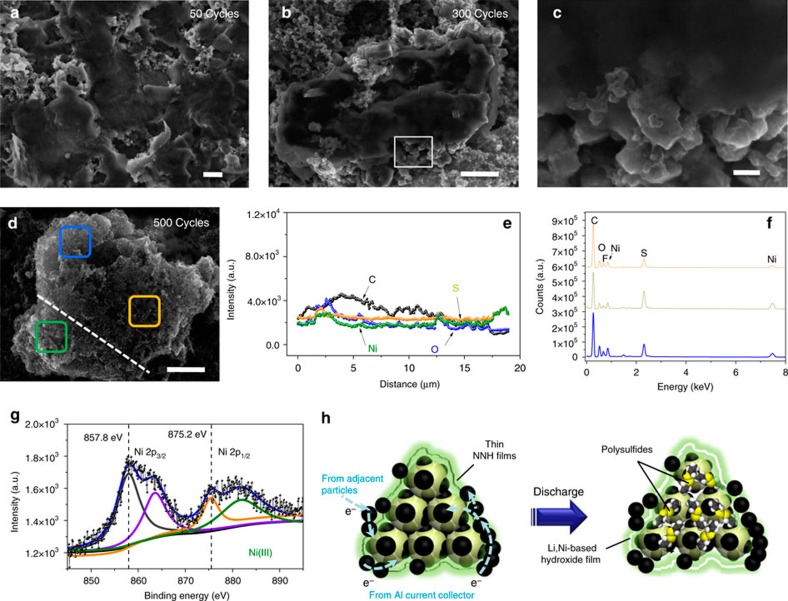
Characterizations of cycled S_8_@CB@NNH cathodes. (**a**–**d**) The *ex situ* s.e.m. monitoring the cathodic morphological changes on increased cyclic numbers. EDX probing towards the S_8_@CB@NNH cathode after 500 cycles: (**e**) a line-scan analysis and (**f**) EDX records on different particle zones. (**g**) High-resolution Ni 2p XPS spectrum and (**h**) a schematic showing working mechanisms of NNH in Li/S cathodes. Proper pathways for electron transfer in the discharge stage have also been displayed. Scale bars, 10 μm (**a**,**b**,**d**) and 100 nm (**c**).

Deep understanding on the working principles of NNH in Li/S cells is made using *ex situ* s.e.m. monitoring coupled with precise EDX probing, Raman spectroscopy, XRD and surface-sensitive X-ray photoelectron spectroscopy (XPS) measurements based on the disassembly of cycled cells at the charge-end state of 50th, 300th and 500th, respectively. Figure 5a–d in turn displays representative s.e.m. images of disassembled cells after different cycles. The top-view s.e.m. image (Fig. 5a) clearly depicts that bulky S_8_@CB@NNH particles remain densely packed and well embedded in the film electrode after 50 times of full charge. In addition, there are layered structures definitely filled in electrode matrices. The electrode suffering from 300 continual cycles looks similar to that in the former case. Though the cathode film to some extent becomes loose possibly due to volume expansions and structural reconfigurations of electrode during lithiation/delithiation, the close encapsulation of protective armors on S_8_@CB particles is always maintained (Fig. 5b). Attentions to morphological features of cycled S_8_@CB@NNH have been paid specially. A zoom-in s.e.m. observation on a selected edge place (Fig. 5c) discloses that the subunits of S_8_@CB@NNH (size: 50∼150 nm, a bit larger than pristine S_8_@CB unit) are still underneath the protection of gel-like film structures despite the situation that cells have run uninterruptedly for hundreds of cycles. The geometric observation on the cathode (Fig. 5d) unambiguously demonstrates that bulks of S_8_@CB@NNH, though turning into porous structures in whole or in part, are able to survive a long-time cyclic test over 500 times charge and discharge (lasting for more than half a year) and still preserve an integrated electrode construction. Line-scan analysis across one cycled S_8_@CB@NNH particle (Fig. 5e) exhibits a uniform elemental distribution of C, S, O and Ni, proving that the functionalized hybrid configurations cannot be varied tremendously even if suffering from long-term input/output operations. EDX testing towards different particle zones (Fig. 5f) give out an identical result, reconfirming the good retention ability on electrode constituents. XRD measurement is used to identify the chemical compositions of cycled S_8_@CB@NNH after 50 cycles (Supplementary Fig. 10a). The presence of a broad diffraction signal instead of original sharp peaks illustrates that the primal S_8_ crystals have evolved into an amorphous state. Moreover, a wide (001) diffraction peak also turns up at 20.4^o^, which is well indexed to the (001) phase of LiOH (JCPDS no. 32-0564). To illustrate LiOH comes from reactions between Li^+^ and NNH, Raman spectroscopy is implemented on cycled electrode of CB@NNH purposely. In Raman spectrum (Supplementary Fig. 10b), we readily distinguish the characteristic peaks of LiOH, which are successively assigned to *A*_*1g*_ mode (329 cm^−1^) and *E*_*g*_ mode (531 cm^−1^ and 620 cm^−1^)^37^, revealing a fact that LiOH is indeed produced after irreversible electrochemical reactions mentioned above. XPS testing is further used to determine the chemical state of Ni in the formed composites coating on S_8_@CB. High-resolution Ni 2p XPS spectrum (Fig. 5g) shows that two prominent peaks (indexed to Ni 2p_3/2_ and Ni 2p_1/2_) are located at binding energies of 857.8 and 875.2 eV, with satellite peaks at high binding energies of 863.5 and 880.4 eV, respectively. All these features have been fully evidenced in the literatures as typical fingerprints of Ni(III) oxidation state (rather than the initial Ni(II) state)^38^. The variation in chemical valence hints that the involved Ni element in armors may also take part in redox reactions, possibly acting as a mediator/catalyst role via interactions with polysulfides to improve the reaction kinetics^40^, and contribute to the cell capacity (thus the total specific capacity is even a bit higher than that of S_8_). To further understand the interactions among pure NNH (exclude CB since CB itself has a reversible capacity when working in cell systems), Li^+^ and polysulfides in Li/S cells, more systematic researches will be highly encouraged and concentrated in our future work via using distinct binders (like CMC, LA132 and so on). The CV comparisons between the S_8_@CB@NNH and S_8_@CB electrodes after 100 cycles (Supplementary Fig. 11) may grant us important hints to account for the capacity exceeding as well as extended plateaus. Clearly, S_8_@CB@NNH exhibits a higher current intensity than S_8_@CB owing to its preferable capacity retention. Notice that except for signal increases at/around peak positions, additional current enhancements are found in other potential regions (for example, from ∼2.0 to 2.3 V) as well. This implies that besides electrochemical reactions of S_8_ (the main capacity contributor), other gentle reversible conversions in cathode systems (typically like pseudocapacitive and inner redox reactions or interactions and so on) may give partial contributions to the ultimate cell capacity.

Above characteristics and evidences guide us to unveil the working mechanism of NNH in Li/S cathodes, as schematically described in Fig. 5h. Among primal tens of cycles, the majority of thin-layered NNHs surrounding the S_8_@CB particles are gradually transformed into more electrochemically stabilized Li-based hydroxide compounds via mild irreversible discharge reactions without causing any structure collapses or film pulverizations. Abundant incorporation of hydrophilic and surface hydroxyl groups that are reported to bind favourably with polysulfide anions^12^,^39^, combined by a good physicochemical stability of such derived substances could effectively assure the suppression of polysulfides dissolution and leakage. This has been testified by good electrochemical properties of S_8_@CB@NNH, with remarkable extended cyclability, greatly enhanced S_8_ utilization and fairly high coulombic efficiencies (all the time retaining ∼98% above). Moreover, the multi-layered armors compactly ‘worn' on S_8_@CB would never deteriorate the ionic transfer property. On one hand, rich Li^+^ in electrolyte phase may enter the inner places of S_8_@CB@NNH by means of Li^+^ exchange with Li, Ni-mixed hydroxides wrapping outside the S_8_@CB. On the other hand, the massive and rapid ionic migrations/diffusions can proceed smoothly throughout effortlessly traversing micropores/mesopores that intrinsically exist on NNH films or *in situ* created by Li^+^ on thin-layered structures during deep charge/discharge cycling.

## Discussion

In this work, the thin-layered Ni-based hydroxide has been demonstrated for the first time as one feasible and effective encapsulation material to prolong the service life of Li/S cells. Our uncustomary selection of hydroxides as an encapsulation candidate is highly triggered by three critical factors as follows:

First and foremost, there exist electrochemically irreversible interactions between Li^+^ and layered hydroxides, leading to the generation of a more stabilized shelly structure. This shelly structure can function as a robust protective layer to entrap the active S_8_ and intermediate polysulfide molecules inside the cathodes, hence efficiently extending the cell cycle lifespan.

Next, our used layered NNH itself is pretty thin. With that, this gauzy covering layer allows the ease of Li^+^ access into the cathode either by diffusions across both interspaces and micropores/mesopores or via Li^+^ exchanges occurring on the solid–liquid interface. This ionic transit is much easier than other cases for thick physical barriers.

Last but never the least, the thin, soft and flexible film prepared in solution phase is apt to worn uniformly on involved precursors no matter what forms and shapes they are present in. This general and simple synthetic strategy, thereby, would pledge the mass production of integrated core–shell hybrid constructions with both intact and intimate wrapping properties.

As a proof-of-concept demonstration, our designed S_8_@CB@NNH hybrid cathode proves great improvements in capacity retention and long-cyclic ability. Unlike previous approaches to trap polysulfides by physical barriers or simple surface interactions, our method is quite efficient, simple, cost-effective and particularly suitable for bulky materials, opening up the prospect of using layered thin-metal hydroxides as a creative and effective class of encapsulation materials for promotions of Li/S cell performance. Despite these advances, in the way still stand formidable challenges, typically like the presence of undesired potential sluggish/polarizations associated with increased charge-transfer resistances due to tight capsulations of NNH. To decrease such polarizations during the cell operation, further development of better thin shells/layers by means of proper and smart engineering/hybridization of NNH with other robust and conductive substances may hold great promise in future Li/S cell technology and deserve our systematic and elaborate studies.

## Methods

### Preparation of S_8_@CB hybrid

The S_8_@CB hybrid was prepared following a melt-diffusion strategy. S_8_ powders (8 g; Sigma-Aldrich, Index-No. 016-094-00-1; assay ≥99.5%) and CB (2 g; LITX 50, Cabot China (Shanghai) Ltd.; ∼45–60 m^2^ g^−1^) were grounded together (the optimal weight ratio of S_8_/CB was found to be 4:1), and then heated to 155 °C and maintained for 6 h.

### Preparation of S_8_@CB@NNH hybrid

The S_8_@CB@NNH hybrid was synthesized by using S_8_@CB hybrids as the starting materials. In details, a mixture of S_8_@CB powders (0.6 g), hexamethylenetetramine/C_6_H_12_N_4_, 0.5 g (Sigma-Aldrich, Index-No. 612-101-00-2; assay ≥99.0%), Ni(NO_3_)_2_·6H_2_O (0.25 g; Sigma-Aldrich, Index-No. 028-012-00-1; assay ≥97.0%) and distilled water (50 ml) was magnetically stirred for 30 min. Afterwards, the resulting suspension was transferred into a sealed container (80 ml) and held at 95 °C for 6 h. Samples were then collected, washed by distilled water several times and dried at 60 °C in electronic oven.

### Characterization

The powder samples were characterized by field-emission SEM (JSM-6700F, 5.0 kV) collocated with a high-precision energy dispersive spectroscopy system (Oxford Instruments of X-Max^N^; its silicon drift detector comes in a wide range of detecting sizes, from 20 mm^2^ for microanalysis up to an astounding 150 mm^2^ for advanced nanoanalysis), XRD (Bruker D8 Advance diffractometer with Cu Kα radiation, *λ*=0.15418, nm) and N_2_ adsorption/desorption (ASAP2020 volumetric adsorption analyser; Micromeritics, USA). The morphology and crystalline structure of NNH were further characterized using a TEM/high-resolution TEM (JEM 2100F). XPS spectrum was measured on a Perkin-Elmer model PHI 5600 XPS system with a resolution of 0.3–0.5 eV from a monochromated aluminium anode X-ray source. Electrical properties were recorded by the use of a Keithley 4200 semiconductor characterization system (USA). The Raman spectrum was made by using a WITec CRM200 Raman system with 532 nm excitation laser.

TG analysis was performed on a SDT600 apparatus under a heating rate of ∼5 K min^−1^ in Ar atmosphere. For the acid immersion treatment, 1 g of fresh S_8_@CB@NNH hybrid powder was put into a 100 ml glass beaker wherein a 60 ml of 3 M hydrochloric acid (HCl) solution was contained. This mixture was then subjected to a 30 min of mild magnetic stirring (rotating rate: 200 r min^−1^) so as to guarantee the complete removal of NNH. The next sample collection procedure is much crucial to the acid immersion treatment. Powders were carefully collected via a slow vacuum filtration process using a microporous membrane filter (Nylon 66; pore size: 0.22 μm), washed with deionized water several times and dried under vacuum conditions at room temperature. To avoid weight losses, nearly all of the residuals on container are thoroughly collected. The powder mass was then determined by a microbalance with an accuracy of 0.01 mg (A&D Company N92, Japan). According to mass differences, NNH statistically took up ∼8.2% in S_8_@CB@NNH hybrids.

### Electrochemical measurements

The working electrodes were all prepared by mixing powder samples with polyvinylidene difluoride (Sigma-Aldrich, Product No. 427152) binder and CB (weight ratio: 80:10:10) in *N*-methyl-2-pyrrolidone (Sigma-Aldrich, assay ≥99%) to form a slurry, which was then pasted onto aluminium foil and dried in an electrical oven. Bare S_8_@CB and CB@NNH electrodes were also made in the same way for electrochemical comparisons. The 2032-type coin cells were then assembled in an Ar-filled glovebox (MBRAUN, UNIlab, Germany) by using Li foil as the counter electrode. The used electrolyte was lithium bis(trifluoromethanesulphonyl)imide (1 M) in 1:1 (v/v) 1,2-dimethoxyethane and 1,3-dioxolane (Fosai New Material Co., Ltd. and Kanagawa Co., Ltd., Japan). LiNO_3_ salt was added (1 wt%) to help passivate the Li anode surface. The mass of electrode materials was measured on a high-accuracy microbalance. The mass ratio of S_8_ in synthesized S_8_@CB@NNH hybrid is ∼78%, while the ratio value in pasted electrodes is ∼62.4% due to additives of CB and polyvinylidene difluoride. For one electrode, the typical mass loading was ∼1.8–2.5 mg cm^−2^ unless otherwise stated. The specific capacity values are calculated based on the total mass of electrode materials (excluding CB and polymer binders). In cell testing, the charge/discharge profiles and cyclability data were obtained with a programmable battery cycler (Neware Instruments). The C rates specified in this case study are based on the mass and theoretical capacity of S_8_ (1C=1,675 mA g^−1^). All cells were aged for 8 h before the cyclic test.

## 

## Additional information

**How to cite this article:** Jiang, J. *et al.* Encapsulation of sulfur with thin-layered nickel-based hydroxides for long-cyclic lithium–sulfur cells. *Nat. Commun.* 6:8622 doi: 10.1038/ncomms9622 (2015).

## Supplementary Material

Supplementary Information Supplementary Figures 1-11
