# Supplementary material for: Encapsulation of sulfur with thin-layered nickel-based hydroxides for long-cyclic lithium–sulfur cells
Source: Nat Commun. 2015 Oct 16;6:8622. doi: 10.1038/ncomms9622 (PMC4634335; doi:10.1038/ncomms9622)
Supplement: Supplementary Information — Supplementary Figures 1-11 [file ncomms9622-s1.pdf]

Supplementary Figures

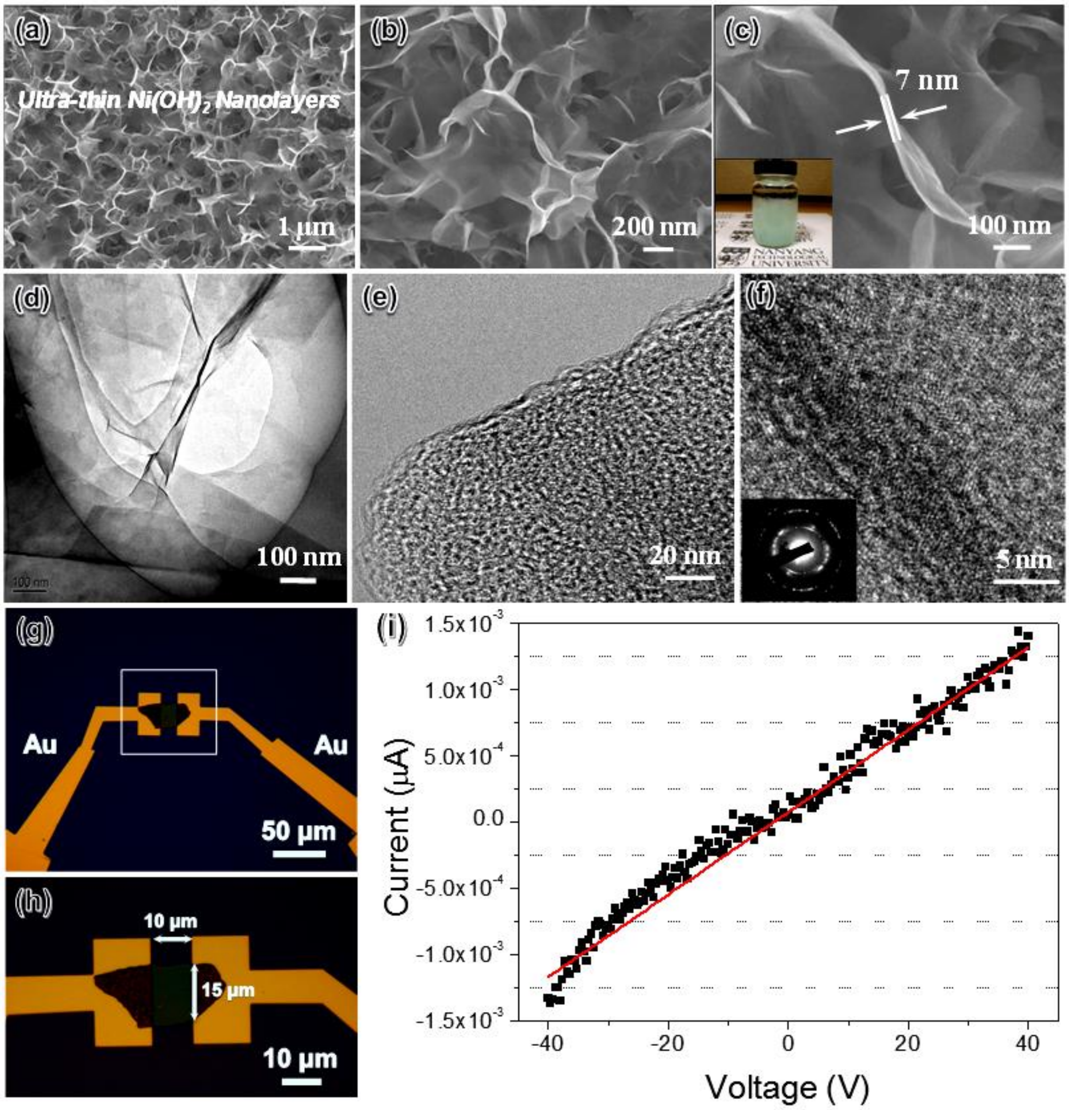

Supplementary Figure 1 (a-c) SEM, (d-e) TEM and (f) HRTEM observations on thin NNH nanolayers. (g-h) Micrographs and (i) *I*-*V* plot of single NNH layer-based device. According to measured parameters and *I*-*V* data, the electrical conductivity of NNH is determined to be ~3.5×10^{-3} s m^{-1}.

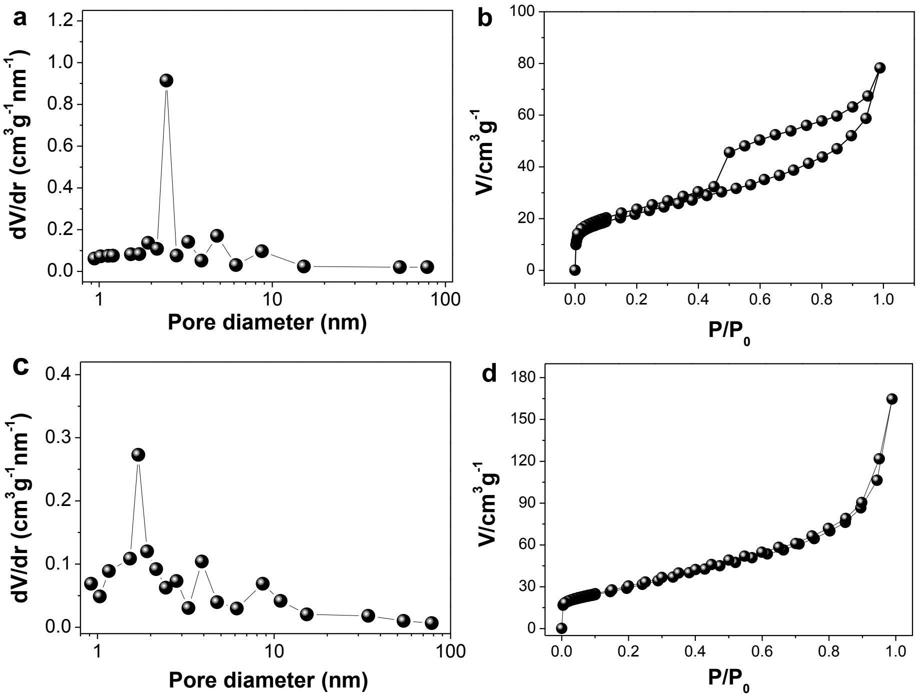

Supplementary Figure 2 (a) Pore-size distribution (PSD) plot and (b) N_2 adsorption isotherm of pure NNH. The specific surface area (SSA) of NNH is measured to be ~54.19 m^2 g^{-1}. (b) PSD plot and (d) N_2 adsorption isotherm of CB powders.

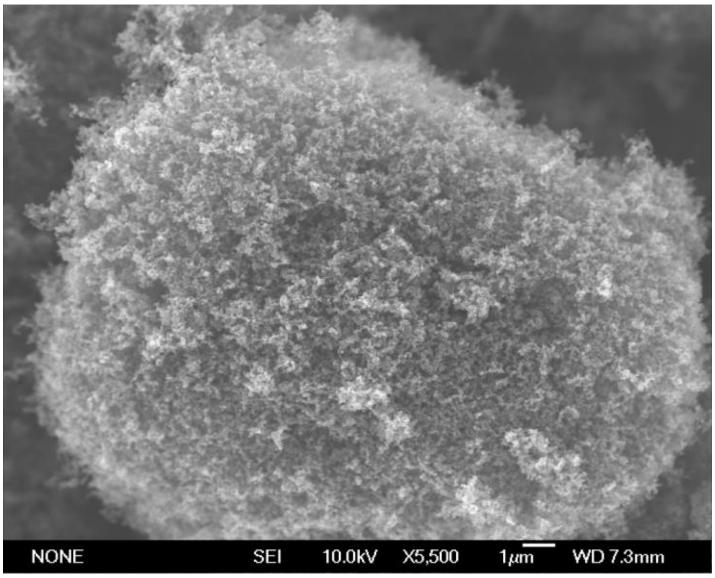

Supplementary Figure 3 SEM image of a bulky S_8@CB particle with a central diameter of ~20 μm.

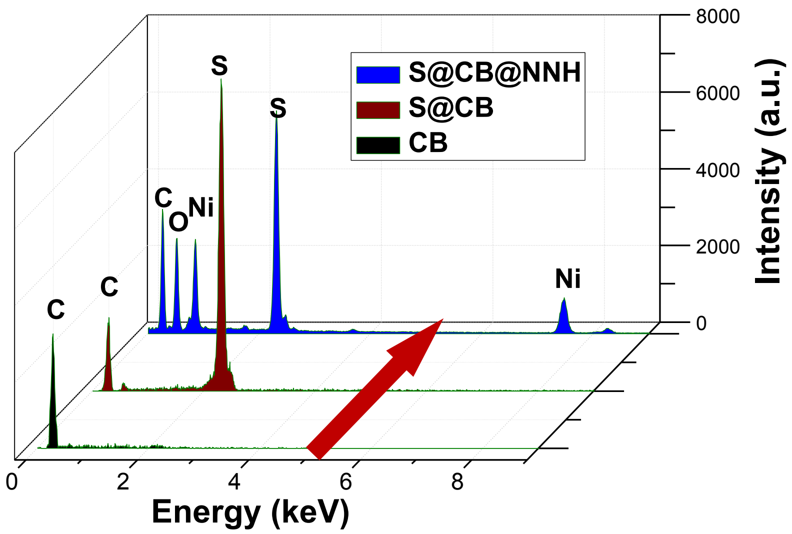

Supplementary Figure 4 EDX spectra gained at different synthesis stages of S_8@CB@NNH.

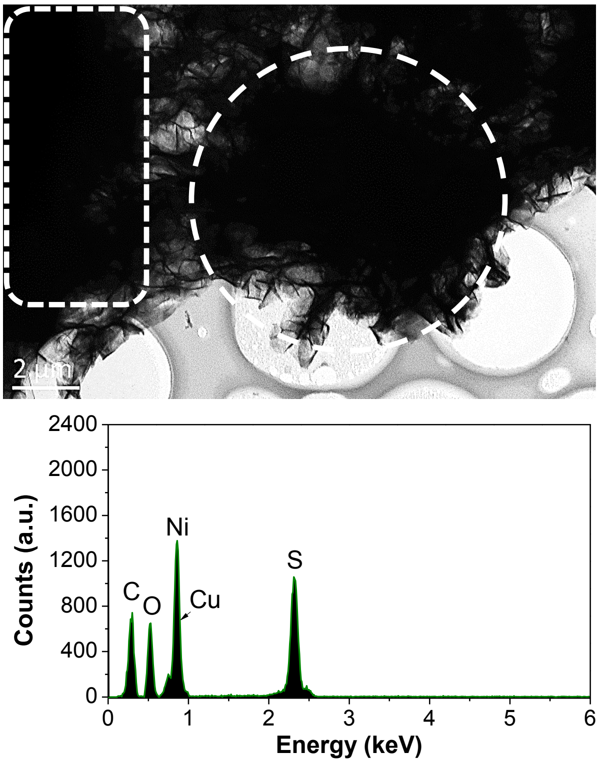

Supplementary Figure 5 TEM image and EDX spectrum of S_8@CB@NNH product.

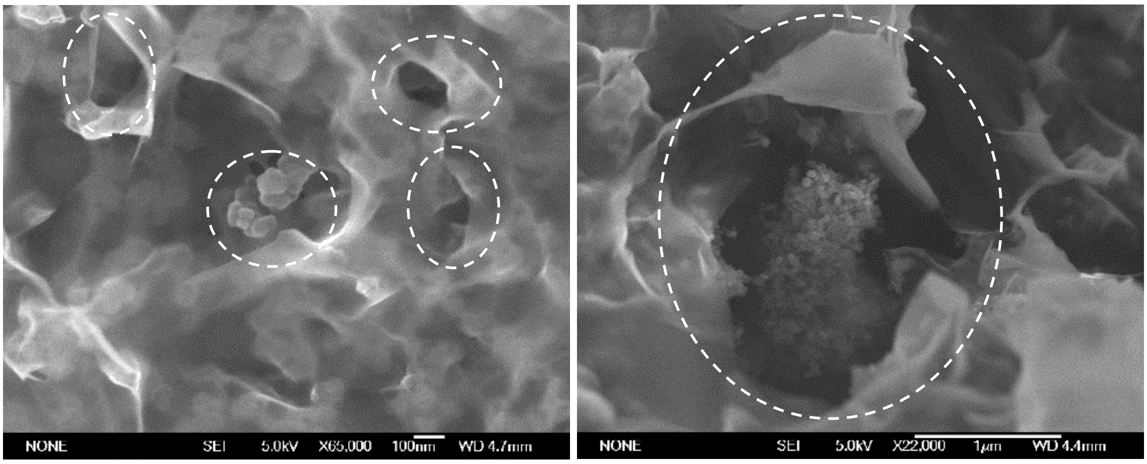

Supplementary Figure 6 High-resolution SEM observations toward S_8@CB@NNH showing the existence of pore defects on the hybrid surface. The formation of such meso-pores on outer surfaces may be due to structural imperfects or breakage/damage of NNH layers during fabrication, whereas those large pores would be designated to interfacial regions between neighboring NNH layers.

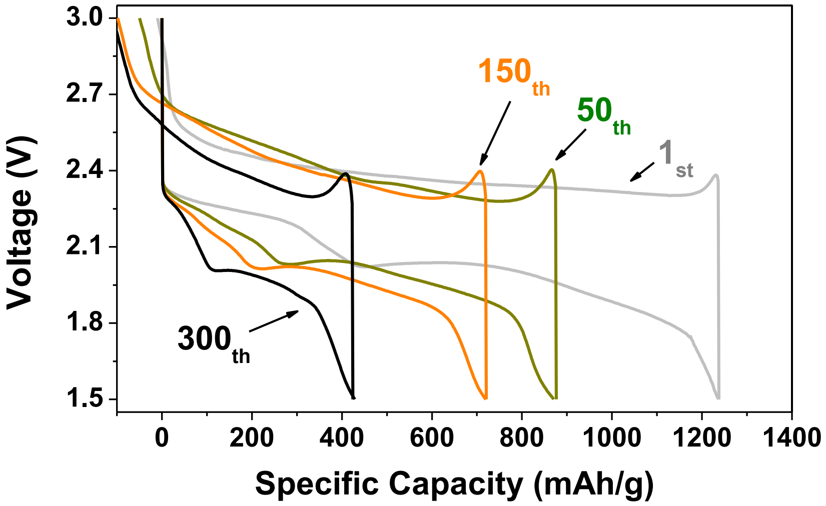

Supplementary Figure 7 Charge/discharge voltage profiles of bare S_8@CB cathode at 0.2C.

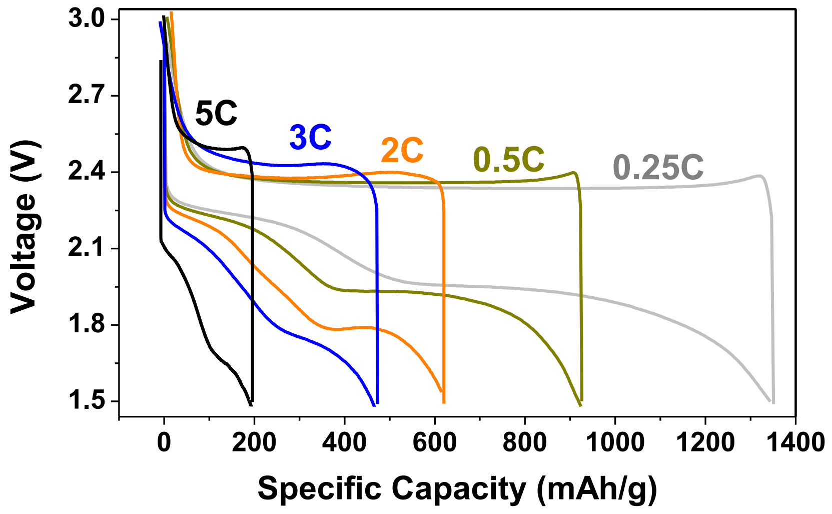

Supplementary Figure 8 Charge/discharge voltage profiles of S_8@CB@NNH cathode performed at varied current rates.

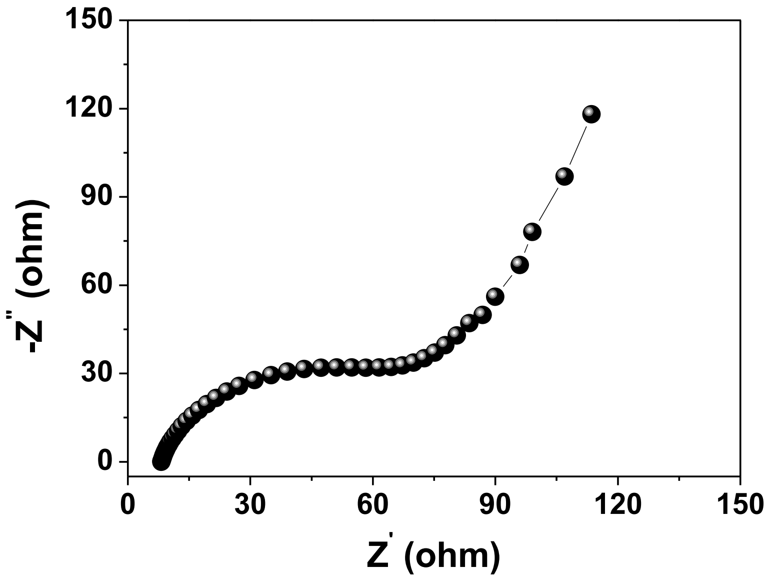

Supplementary Figure 9 Electrochemical impedance spectrum of bare S_8@CB cathode.

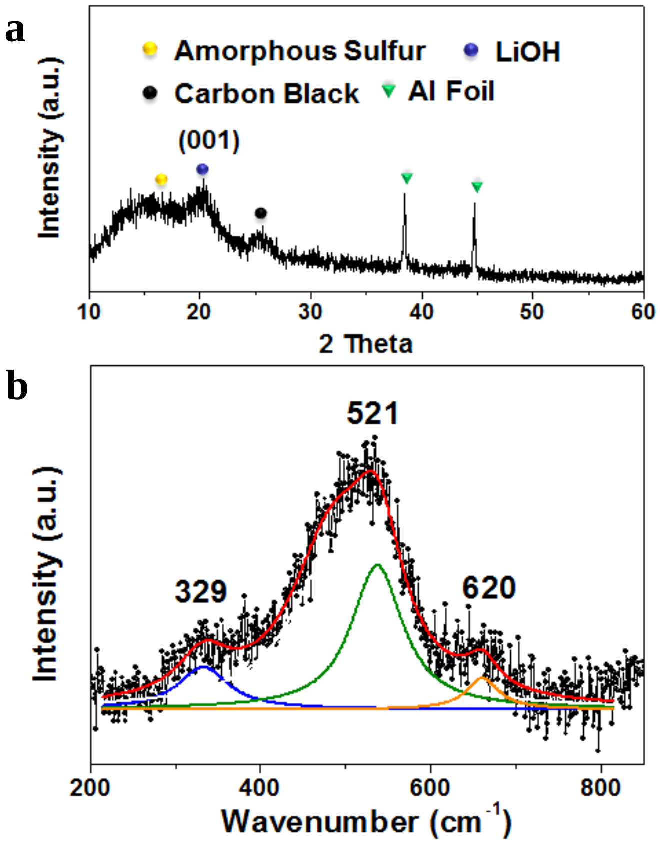

Supplementary Figure 10 (a) XRD pattern of cycled S_8@CB@NNH cathode after 50 cycles. (b) Raman spectrum of cycled CB@NNH electrode.

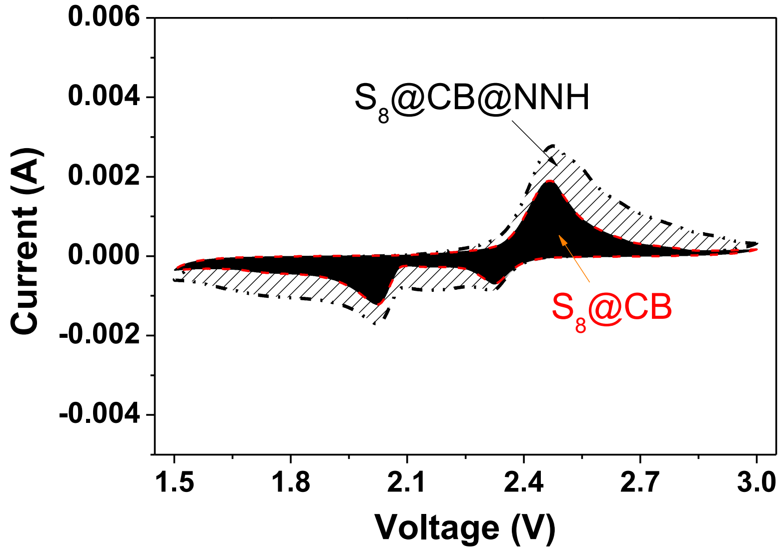

Supplementary Figure 11 CV curves of the S_8@CB@NNH and S_8@CB electrodes after 100 cycles under a same current rate of 0.5C.
